# Principles of Human Physiology; with Their Chief Applications to Pathology, Hygiene, and Forensic Medicine

**Published:** 1850-02

**Authors:** 


*Principles of Human Physiology; with their chief applications to Pathology, Hygiene, and Forensic Medicine.* By William B. Carpenter, M. D., F. R. S., F. G. S., etc., etc., etc. Fourth American Edition, with extensive additions and improvements by the author. With two plates, and three hundred and four wood cuts. Philadelphia: Lea & Blanchard. 1850. 8vo., pp. 750.

Our opinion of the merits of Dr. Carpenter's writings on Physiology has already been expressed in notices of former editions of this work. Suffice it to say, now, that the medical pupil and practitioner who would study the science of Life in its present position, can hardly dispense with Dr. Carpenter's productions. The present edition of his principles of Human Physiology has been prepared with express reference to the American reader; that is to say, he has been induced to supply extensive additions and improvements for this edition, at the instance of the enterprising and liberal publishers from whose house the work emanates. It is but justice to the publishers and to the author, as well as to our readers, to submit some account of the new matter contained in this edition. For this end we copy the following from the Preface:—

It has been with no small gratification, that the Author of the following Treatise has been apprized of the estimation in which it is held by the medical public of the United States, as evinced by the rapid sale of large impressions of the American reprint of each successive English edition. In the present instance, the demand for a new issue in the United States has anticipated the exhaustion of the last English edition; and the American publishers must have therefore contented themselves with simply reprinting the work, many parts of which are now behind the present state of Physiological knowledge (so rapid is the progress of the science), if they had not adopted the wiser course of making it worth the Author's while to prepare a new Edition specially for their press. The Author has, therefore, the opportunity of laying before his American readers his latest views on several topics which have constantly been occupying his own attention, together with the results of the recent labours of other inquirers on numerous points of interest.

The principal changes will be fonnd in the portion of the work which treats of the Nervous System; and in the Chapter on Generation. The former have been rendered necessary by the progress of the Author's own inquiries, which have led him to relinquish certain parts of Dr. Marshall Hall's doctrines long advocated by himself, and to substitute what he believes will be found a far simpler and more consistent view of the constitution of the Cerebro-spinal centres, which may be said to be essentially based on the doctrines of Messrs Todd and Bowman, though differing from them in some important particulars. Those who may be interested in seeing some of these questions discussed more fully and critically than the scope and character of this work permit, are referred to the British and Foreign Medico-Chirurgical Review for January, 1850. The alterations in the Chapter on Generation have chiefly consisted in the substitution of the views of Bischoff, with respect to the development of the ovum, for those of Dr. Barry; the Author having satisfied himself that the latter are no longer tenable, whilst the statements of the former, though imperfect in many important particulars, are, on the whole, deserving of the credit which he had previously accorded to Dr. Barry's observations.

Numerous other additions and alterations, which, taken collectively, constitute no inconsiderable amount of matter, are scattered through the Treatise; their importance being greatest, however, in the Chapter on the Primary Tissues, and in that on Nutrition. The Author has done his best, in the limited time permitted him for the prepararion of the Edition —that time being already muce occupied by other engagements, and having been still further curtailed by domestic anxieties—to render this edition deserving, in a full measure, of the favor which its predecessors have experienced from the medical public of the United States; and he ventures to say of it, as he did of the preceding, that "he trust he may be found to have generally exercised a sound discretion, both as to what he has admitted, and what he has rejected; and that his work will appear to exhibit, on the whole, a faithful reflection of the present aspect of Physiological Science."

By the liberality of the Publishers, a considerable number of additional wood cuts, the subjects of nearly all of which are new, have been introduced into this Edition; and one of the lithographic plates has been replaced by another of more accurate character and superior execution. The number of *new* wood cuts is considerably greater than would be indicated by the mere difference in the total amount between those of the last edition and those of the present; several of the least important among the former having been omitted to make way for them, in order that the bulk of the volume might not be too much augmented.

